# Sub-national tailoring of seasonal malaria chemoprevention in Mali based on malaria surveillance and rainfall data

**DOI:** 10.1186/s13071-022-05379-4

**Published:** 2022-08-04

**Authors:** Mady Cissoko, Issaka Sagara, Jordi Landier, Abdoulaye Guindo, Vincent Sanogo, Oumou Yacouba Coulibaly, Pascal Dembélé, Sokhna Dieng, Cedric S. Bationo, Issa Diarra, Mahamadou H. Magassa, Ibrahima Berthé, Abdoulaye Katilé, Diahara Traoré, Nadine Dessay, Jean Gaudart

**Affiliations:** 1 Malaria Research and Training Centre Ogobara K. Doumbo (MRTC-OKD), FMOS-FAPH, Mali-NIAID-ICER, Université Des Sciences, Des Techniques Et Des Technologies de Bamako, 1805 Bamako, Mali; 2 INSERM, IRD, ISSPAM, UM1252, Aix-Marseille University, 13005 Marseille, France; 3 Direction Régionale de la Santé de Tombouctou, 59 Tombouctou, Mali; 4 Programme National de Lutte contre le Paludisme (PNLP Mali), 233 Bamako, Mali; 5 Direction Générale de la Santé et Hygiène Publique, Sous-Direction Lutte Contre la Maladie (DGSHP-SDLM), 233 Bamako, Mali; 6 ESPACE-DEV, UMR228, IRD/UM/UR/UG/UA, Institut de Recherche Pour le Développement (IRD) France, 34093 Montpellier, France; 7 APHM, INSERM, SESSTIM, ISSPAM, Hop Timone, BioSTIC, Biostatistic & ICT, Aix-Marseille University, 13005 Marseille, France

**Keywords:** Malaria, High transmission season, Rainfall, Sub-national, Tailoring

## Abstract

**Background:**

In malaria endemic countries, seasonal malaria chemoprevention (SMC) interventions are performed during the high malaria transmission in accordance with epidemiological surveillance data. In this study we propose a predictive approach for tailoring the timing and number of cycles of SMC in all health districts of Mali based on sub-national epidemiological surveillance and rainfall data. Our primary objective was to select the best of two approaches for predicting the onset of the high transmission season at the operational scale. Our secondary objective was to evaluate the number of malaria cases, hospitalisations and deaths in children under 5 years of age that would be prevented annually and the additional cost that would be incurred using the best approach.

**Methods:**

For each of the 75 health districts of Mali over the study period (2014–2019), we determined (1) the onset of the rainy season period based on weekly rainfall data; (ii) the onset and duration of the high transmission season using change point analysis of weekly incidence data; and (iii) the lag between the onset of the rainy season and the onset of the high transmission. Two approaches for predicting the onset of the high transmission season in 2019 were evaluated.

**Results:**

In the study period (2014–2019), the onset of the rainy season ranged from week (W) 17 (W17; April) to W34 (August). The onset of the high transmission season ranged from W25 (June) to W40 (September). The lag between these two events ranged from 5 to 12 weeks. The duration of the high transmission season ranged from 3 to 6 months. The best of the two approaches predicted the onset of the high transmission season in 2019 to be in June in two districts, in July in 46 districts, in August in 21 districts and in September in six districts. Using our proposed approach would prevent 43,819 cases, 1943 hospitalisations and 70 deaths in children under 5 years of age annually for a minimal additional cost. Our analysis shows that the number of cycles of SMC should be changed in 36 health districts.

**Conclusion:**

Adapting the timing of SMC interventions using our proposed approach could improve the prevention of malaria cases and decrease hospitalisations and deaths. Future studies should be conducted to validate this approach.

**Graphical Abstract:**

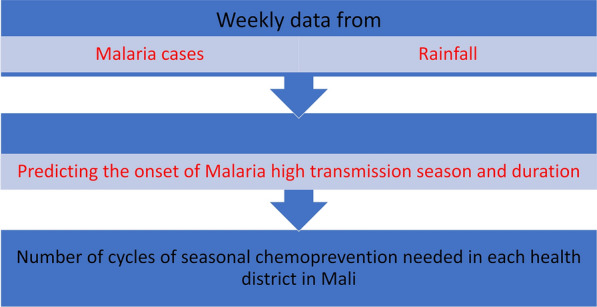

## Background

In 2020, 482,790 deaths attributable to malaria were reported in children under 5 years of age worldwide, and 95% of the 242 million cases of malaria occurred in Africa [1]. In Mali, the Local Health Information System recorded 1454 hospital deaths attributable to malaria out of 2,884,837 confirmed cases in 2020. The 2018 Mali Demographic and Health Survey (DHS) reported a mortality of 101 per 1000 children [2], a figure considerably higher than the average mortality of 76 per 1000 children reported in 2019 by the World Bank for the Sub-Saharan Africa region [3].

The WHO technical guidelines for malaria control recommend the implementation of targeted interventions in malaria endemic countries [4]. Targeting consists of tailoring interventions to the local epidemiological context to make better use of available financial resources [5, 6]. Recommended interventions include community-based diagnostic and treatment, chemoprevention for children aged 3–59 months and pregnant women as well as vector control using long-lasting insecticidal nets (LLINs), indoor residual spraying (IRS) and larviciding [4, 7]. In low-prevalence countries (i.e. malaria prevalence < 1%), mass drug administration is recommended to reduce asymptomatic carriage and to control epidemics [8]. Epidemiological surveillance allows the timing of outbreaks to be determined, epidemics to be detected and the development of parasite and mosquito resistance to be tracked, with the view to adapt the timing of interventions and to check the progress made [4]. Between 2010 and 2017, targeted control interventions helped to reduce the global incidence of malaria by > 40% [9].

In Mali, the multiplication of chloroquine resistance outbreaks prompted the National Malaria Control Program (NMCP) to revise its malaria management protocol in 2005 and then again in 2020 by integrating the new WHO recommendations. Since June 2006, uncomplicated malaria cases are treated with artemisinin-based combination therapy (ACT) and severe malaria cases with injectable artesunate, artemether or quinine (as a last resort) for a period of 7 days, with these injectables being replaced with ACT if the patient’s condition improves rapidly. In places where the technical infrastructure does not allow the continuation of treatment for severe malaria, pre-referral treatment is recommended to reduce the risk of death. Diagnosis and treatment are provided in public and para-public health facilities: rapid diagnostic tests (RDTs) are free for all patients with suspected malaria, and treatment is free for pregnant women and children under 5 years of age [10].

In accordance with WHO recommendations, malaria prevention in Mali involves vector control using LLINs and IRS and chemoprevention in children and pregnant women [10]. A major control intervention is seasonal malaria chemoprevention (SMC) [11], which consists of the monthly administration of antimalarial drugs to children aged 3–59 months during the high malaria transmission season [12]. Since 2016, the NMCP has been implementing SMC interventions in all health districts on the basis of historical national data on malaria incidence or prevalence [13]. Together with financial partners, the NMCP carries out macro-planning in November to budget the costs of the following year’s campaign based on available resources (purchase and transport of SMC drugs). Between May and June, the heads of health districts come together in regional micro-planning workshops to budget the costs of administering SMC at the operational scale (per diem, logistics, supervision, paperwork). Interventions are performed in four cycles of treatment during the high transmission season (from July to November) [13]. Antimalarial drugs (sulfadoxine-pyrimethamine and amodiaquine) are administered by community health workers to children aged 3–59 months either door-to-door or at fixed sites in town centres. Importantly, SMC interventions are increasingly cost-effective [14]: the average unit cost (per child and per cycle) went from 1.1 euro (€) (745 CFA franc) in 2012 [15] to 0.7€ (425 CFA franc) in 2014 [14]. (Note that this unit cost covers drugs, transport, per diems and logistics.)

The efficacy of SMC interventions in Mali has been demonstrated at the health district scale. An 80% reduction in uncomplicated and severe cases has been reported in the health district of Kati (in southern Mali) following the implementation of SMC [13]. One study reported a 61% difference in mortality between health districts with and without SMC interventions [14]. Another study found that deaths and hospitalisations attributable to malaria were 44% and 61% lower, respectively, in health districts where SMC was implemented compared to those where it was not [18]. However, no decline in malaria incidence was observed at the national scale in children under 5 years of age between 2018 and 2020, even though the effectiveness of control interventions did not seem to be compromised by parasite resistance [9], suggesting a need for improving malaria control in Mali, including by tailoring the timing of SMC interventions to that of malaria transmission at the operational scale.

Malaria transmission depends on a combination of meteorological and climatic factors that determine the distribution and abundance of the disease vector* Anopheles* species. In Mali, the main vectors of malaria transmission are *Anopheles gambiae* sensu stricto, *An. funestus*, *An. coluzzii* and *An. arabiensis* [19]. These species share a similar life-cycle: after a period of aestivation [19, 20], they start laying eggs to increase their population as environmental conditions become favourable again (rainy season and irrigation). They then ingest, develop and transmit the plasmodium during their blood meal on asymptomatic human carriers [21]. In accordance with this life-cycle, the number of malaria cases increases after the onset of the rainy season, with a lag that corresponds to the delayed impact on transmission of the increase in mosquito activity [22, 23]. Across the country, the rainy season lasts from April to October, and the peak of malaria transmission occurs in September, with the lag between the onset of the rainy season and that of the high transmission season ranging from 5 to 18 weeks depending on the eco-climatic zone [22, 23]. However, in some of the northern regions, the rainy season lasts from July to October and is followed by a flooding season that lasts from November to February. A second peak usually occurs in December in these regions [24] as transmission can persist for several weeks in flooded areas (sometimes until February) due to the lasting presence of humid zones, aquatic vegetation and the malaria vector species *An. funestus* [24]. These data suggest important spatio-temporal heterogeneity of malaria transmission in Mali, a phenomenon that should be taken into consideration to improve SMC interventions in a context of limited resources. However, while this heterogeneity has been explored in specific regions of Mali [25–27], it has yet to be described at the scale of the entire country.

This study proposes a predictive approach for tailoring the timing and number of cycles of SMC in all health districts of Mali based on sub-national epidemiological surveillance and rainfall data. Our primary objective was to select the best of two approaches for predicting the onset of the high transmission season at the operational scale. Our secondary objective was to evaluate the number of malaria cases, hospitalisations and deaths in children under 5 years of age that would be prevented annually and the additional cost that would be incurred using the best approach.

## Methods

### Study site

Mali is a landlocked country with a surface area of 1,241,248 km^2^. Its population was estimated at 22 million in 2021 (National Population Directorate (DNP) [28].

The country is split into four ecoclimatic zones: the Saharan zone in the north (annual rainfall: < 200 mm), the vast Sahelian zone in the centre (annual rainfall: ≤ 800 mm), the Sudanese-Sahelian zone in the southeast (annual rainfall: 1000 mm) and the Sudanese-Guinean zone in the extreme south (annual rainfall: > 1000 mm). There are two seasons in Mali: the dry season from November to March and the rainy season from April to October. The first rain events occur as intermittent thunderstorms around April or May.

Mali is divided into 75 health districts that correspond to the operational scale of SMC interventions (i.e. micro-planning of activities). Each health district comprises one hospital and 10–20 health areas that provide primary care and conduct epidemiological surveillance.

### Data and data source

#### Malaria data

In Mali, malaria cases are usually confirmed with RDTs in public, para-public and private health facilities at the community level or in hospital. As *Plasmodium falciparum* is responsible for 98% of malaria cases in the country, the RDTs currently in use are based on the detection of *P. falciparum* histidine-rich protein 2 (PfHRP-2) [29, 30]. Malaria cases are occasionally confirmed in hospital by microscopy examination of thick blood smears.

Data on malaria (suspected, tested and confirmed cases as well as deaths) are collected weekly at the health district level by health facility agents (community health workers, technical directors of community health centres, private institutions and hospital data managers) via the epidemiological surveillance system. The data are then reported to the NMCP and entered in the District Health Information Software version 2 (DHIS2) data system.

This study used weekly aggregated data on malaria cases, hospitalisations and deaths for the 75 health districts of Mali over the 2014–2019 period. No personal data were used.

#### Rainfall data

In Mali, 24 meteorological, climatological and agro-bio-climatological stations and 17 synoptic stations are currently in operation [31]. As these stations do not cover all 75 health districts, we used remote sensing rainfall data in our analyses. Weekly rainfall data by health district were extracted from the Climate Hazards Group InfraRed Precipitation with Station data (CHIRPS) rainfall dataset via the Google Earth engine interface [32, 33] for the 2014–2019 period.

#### Population data

Data on population by health district for the 2014–2019 period were obtained from the DNP. These data are updated yearly through multiplying population figures from the General Population and Housing Census of 2009 (GPHC 2009) by the annual population growth rate [34].

#### Intervention cost data

The average unit cost of SMC interventions (per child and per cycle) was obtained from the study by Diawara et al*.* [14]. This cost covers drugs, transport, per diems and logistics, but does not cover health infrastructure.

### Data validation

Data on malaria are monitored weekly and monthly at the local level by a data manager. Feedback is provided to the health facility agents who entered th data into the DHIS2 data system.

With the support of the US President’s Malaria Initiative (USAID and PMI Measure Malaria) [35], the NMCP organises bi-annual reviews of surveillance data in problem areas as part of quality improvement of malaria data (https://pnlp.ml). These reviews are accompanied by formative supervision for all health personnel involved in malaria control and management. Data are then processed and validated at the local, regional and national levels during a yearly workshop involving data management officers, funding partners and hospital and programme representatives [36].

### Data analysis

In the first part of the analysis, we determined the median onset of the rainy season, the median onset of the high transmission season, the median lag between these two events and the median duration of the high transmission season in each health district for the 2014–2019 period.

We determined the median onset of the rainy season in each health district for the 2014–2019 period using weekly rainfall data. Adapting the definition proposed by Sivakumar et al. [37], we defined the onset of the rainy season as the week when rainfall reached 20 mm after 1 April.

The median onset and median duration of the high transmission season in each health district for the 2014–2019 period were determined by performing a change point analysis of weekly incidence data [38, 39]. For each health district, incidence per 1000 person-weeks was calculated by dividing the number of weekly cases by population × time.

The lag between the median onset of the rainy season and the median onset of the high transmission season reflected the delayed impact on malaria transmission of the increase in mosquito activity after aestivation. It was calculated as follows (Eq. 1):

1 $$\text{Median lag}=\text{median onset of high malaria transmission season}-\text{median onset of rainy season}$$

Two predictive approaches were evaluated. The first approach, App-A, predicted the onset of the high transmission season in 2019 using median rainfall data and median lag data for the 2014–2018 period. The second approach, App-B, predicted the onset of the high transmission season in 2019 using rainfall data for 2019 and median lag data for the 2014–2018 period.

The two approaches were evaluated by calculating the difference in weeks between the observed and predicted onset of the high transmission season in 2019. A difference between - 2 and + 2 weeks was considered acceptable. For each health district *i*:

2 $${\text{Error}}_i={\text{observed onset}}_i\text{of high transmission season}-{\text{predicted onset}}_i\text{of high transmission season}$$

The two approaches were also evaluated by calculating the standardised score (*z*) of the onset of the high transmission season for each health district [40]:

$$z = \frac{x - \mu }{{\sigma { }}}$$

where *x* is the predicted value of the onset of the high transmission season; *μ* is the mean value of the onset of the high transmission season in 2014–2019; and *σ* is the standard deviation of the onset of the high transmission season in 2014–2019.

The onset of the high transmission season was used to determine the best start date of SMC interventions in each health district. The duration of the high transmission season was used to determine the number of cycles of SMC needed in each health district.

Incidence data were presented in a box and whisker format. Health districts with irregular seasonality and/or low seasonal transmission were identified based on incidence figures and the results of the change point analysis.

The second part of the analysis determined the number of malaria cases, hospitalisations and deaths in children under 5 years of age that would be prevented annually using the best of the two predictive approaches.

The additional cost of using the best of the two approaches was also estimated. This cost was the difference between the global cost of the current SMC intervention and the global cost that would be incurred using the best approach. Global costs were calculated through multiplying the average unit cost [14] by the number of children per cycle (as validated in the micro-plans).

### Software and packages

All statistical analyses were performed using R software version 3.4 ® Development Core Team, R Foundation for Statistical Computing, Vienna, Austria) packages {Changepoint}{ggplot}.

## Results

### Prediction of the onset of the high malaria transmission season

Over the 2014–2019 period, mean annual rainfall in Mali was 774 mm, varying significantly between health districts, from 60 mm in the north (Taoudenit region in the Saharan zone) to 1384 mm in the south (Sikasso region). The median onset of the rainy season ranged from week (W) 17 (W17; April) in the south (Sikasso region) to W34 (August) in the north (Taoudenit region).

Mean annual malaria incidence over the 2014–2019 period was 90 cases per 1000 person-years, ranging from one case per 1000 persons-years (Taoudenit region) to 285 cases per 1000 person-years (Sikasso and Koulikoro regions). The median onset of the high transmission season ranged from W25 (June) to W40 (October) depending on the health district.

The median lag between the onset of the rainy season and the onset of the high transmission season ranged from 5 to 12 weeks depending on the health district.

**Fig. 1:**
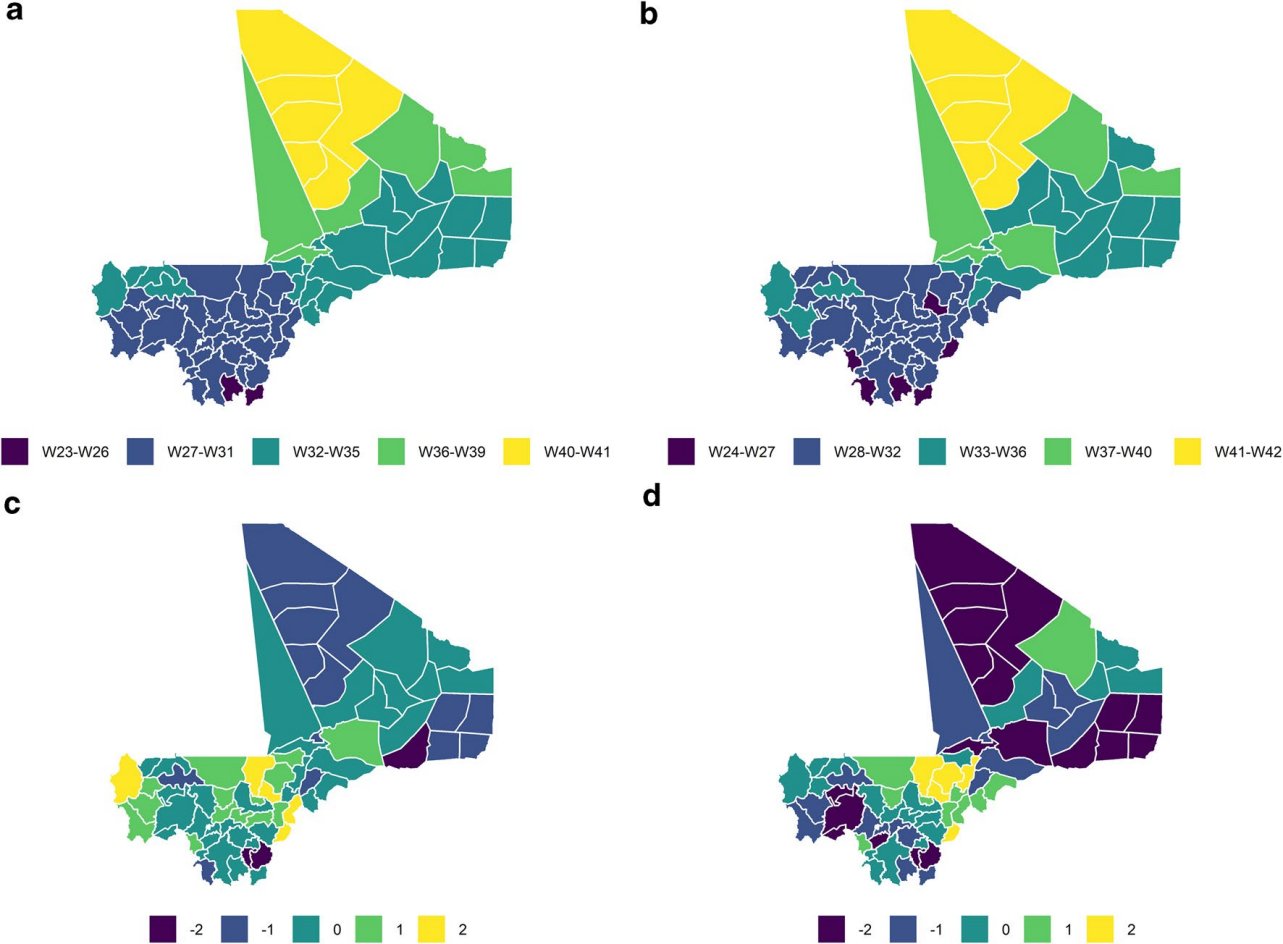
**a**,** b** Maps showing the predicted onset of the high transmission season in 2019, with the colour scale representing the week (W) of onset: **a** predicted onset using the App-A predictive approach (based on median rainfall data and median lag data for the 2014–2018 period, **b** predicted onset using the App-B predictive approach (based on rainfall data for 2019 and median lag data for the 2014–2018 period).** c**,** d** Maps showing the prediction error in weeks, with the colour scale representing the error value (note: a difference between - 2 and + 2 weeks was considered acceptable): **c**prediction error using App-A, **d** prediction error using App-B

Using the first approach (App-A), the predicted onset of the high transmission season in 2019 ranged from W23 (June) to W40 (September). The predicted onset was in June in two health districts, in July in 46 health districts, in August in 21 health districts and in September in six health districts (Fig. 1a).

Using the second approach (App-B), the predicted onset of the high transmission season in 2019 ranged from W24 (June) to W41 (September). The predicted onset was in June in one health district, in July in 43 health districts, in August in 15 health districts and in September in 16 health districts (Fig. 1b).

Both approaches predicted the onset of the high transmission season with an error of ± 2 weeks.

App-A had the best prediction results, with a median error of 0 weeks. No error was found in 34 health districts (45%) and an error of 2 weeks was observed in three health districts (Ansongo, Sikasso and Niena) (4%) (Table 1; Fig. 1c).

**Table 1:** Classification of health districts according to observed and predicted months of onset of the high malaria transmission season in 2019

Month of onset of the high transmission season	Number of health districts according to observed month of onset	Number of health districts according to predicted month of onset using App-A^a^	Number of health districts according to predicted month of onset using App-B^a^
May	4	2	1
June	36	40	43
July	21	21	15
August	14	12	10
September	0	0	6

^a^App-A and App-B are the predictive approaches used in the study. App-A predicted the onset of the high transmission season in 2019 using median rainfall data and median lag data for the 2014–2018 period. App-B predicted the onset of the high transmission season in 2019 using rainfall data for 2019 and median lag data for the 2014–2018 period

App-B showed a median error of 1 week. An error of 2 weeks was observed in the health districts with strong epidemic potential, most of which were located in the northern regions (Ménaka and Taoudenit regions) and in the District of Bamako (Table 1; Fig. 1d).

**Fig. 2:**
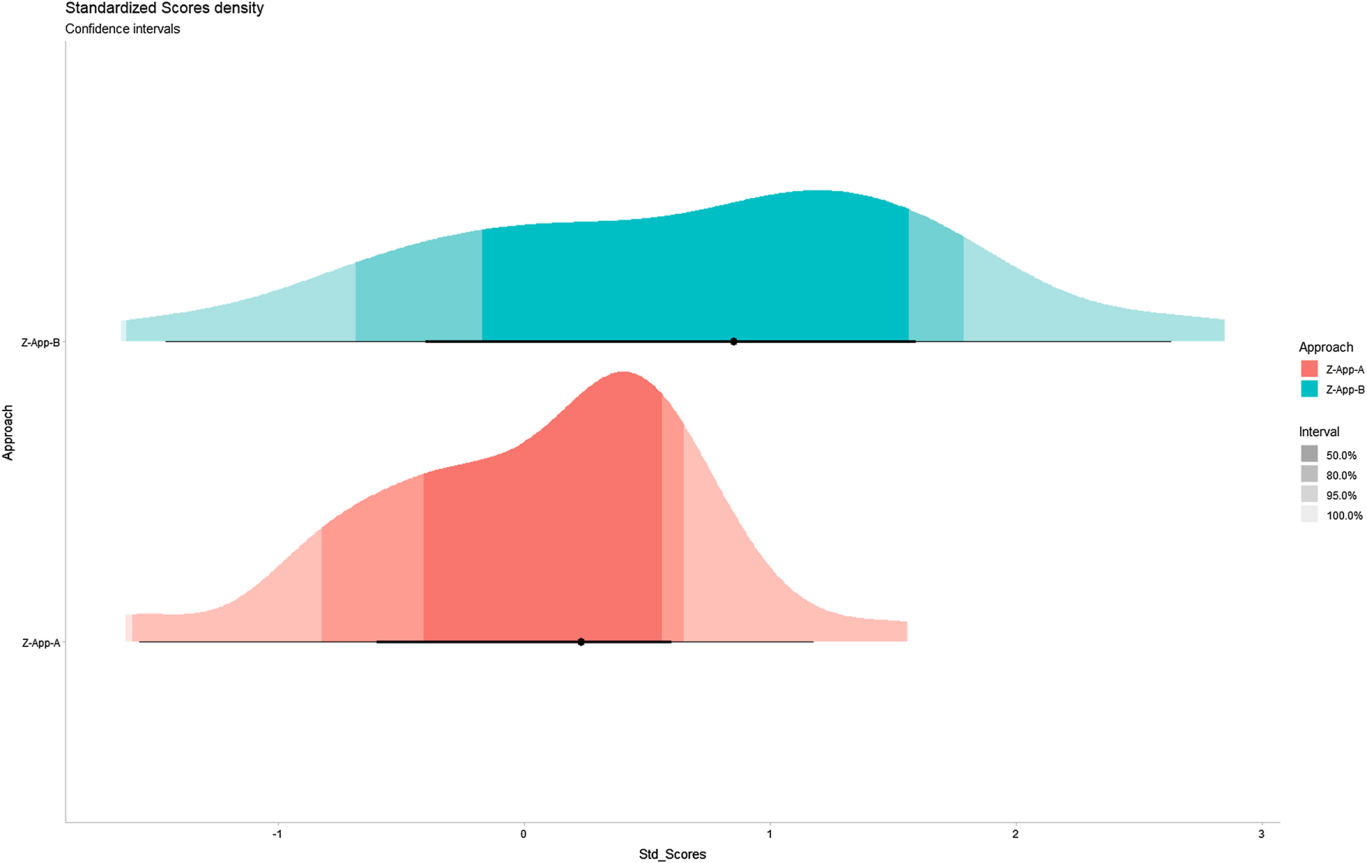
Density of standardised scores with confidence intervals. Standardised scores for App-A and App-B are shown in red and blue, respectively

Both approaches showed high standardised scores. These ranged from a minimum of - 1 to a maximum of + 1 (Fig. 2).

### Duration and seasonality of malaria transmission

**Fig. 3 The Blue line on a silver background represents the smoothing curve and the confidence intervals:**
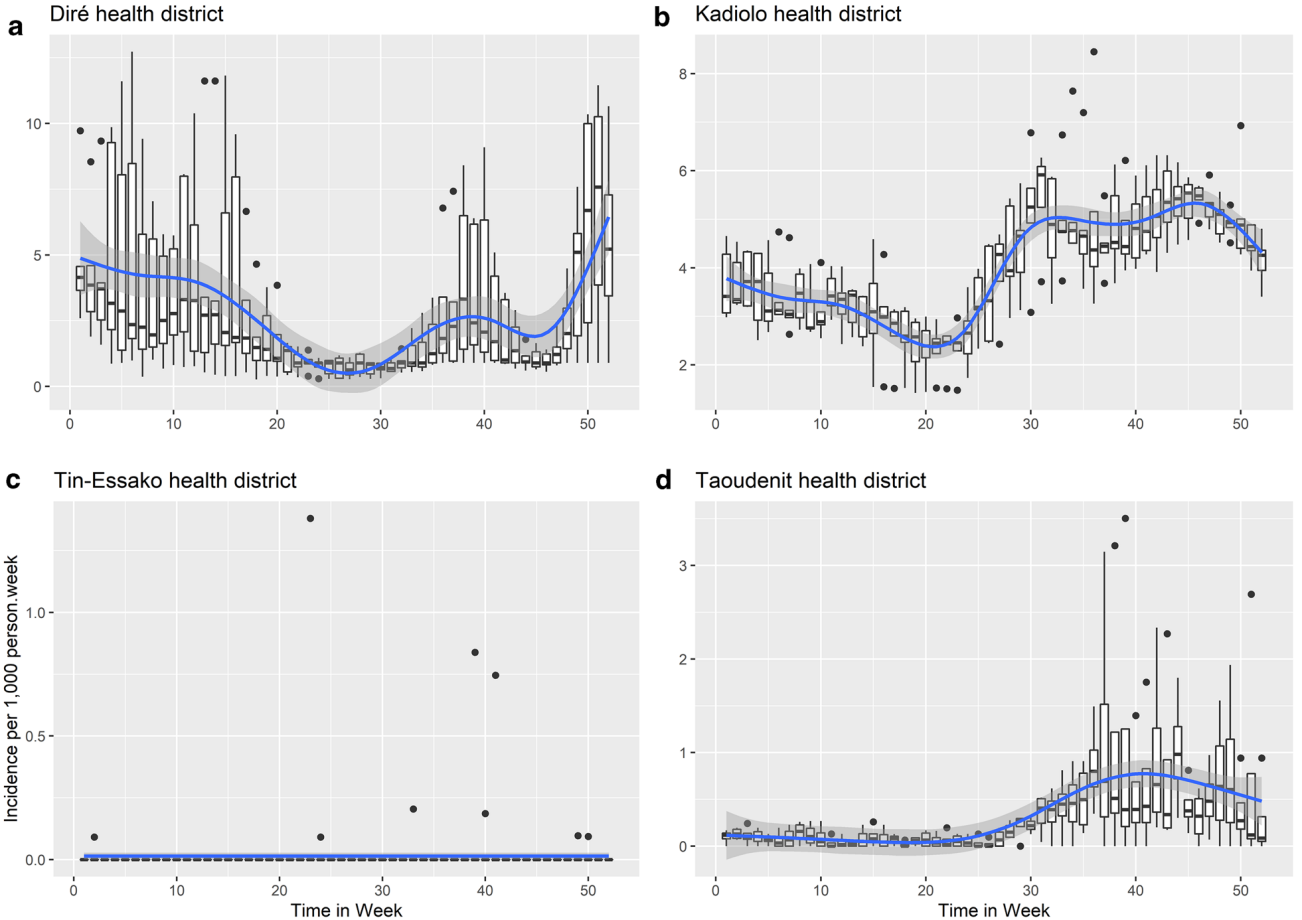
Duration and seasonality of malaria transmission in four representative health districts. **a** Bimodal seasonality, **b** usual seasonality (from July to December), **c** irregular seasonality, **d** low seasonal transmission

Over the 2014–2019 period, the mean duration of the high transmission season was 4 months, and the median duration ranged from 3 to 6 months (Fig. 3a). The high transmission season began between June and August and ended between October and February/March.

Usual seasonality of malaria transmission was observed in 73 health districts (97.33%). Seasonality was bimodal in eight health districts (Ansongo, Diré, Goundam, Gourma Rharous, Menaka, Niafunké, Timbuktu and Youwarou), all of which are located along the Niger river (Fig. 3b). Seasonality was irregular in the health districts of Tinessako, Tessalit and Tindermene (Fig. 3c). In the six health districts of Taoudenit region, seasonal transmission was very low, with a weekly incidence of < 1 case per 1000 person-weeks (Fig. 3d).

**Fig. 4:**
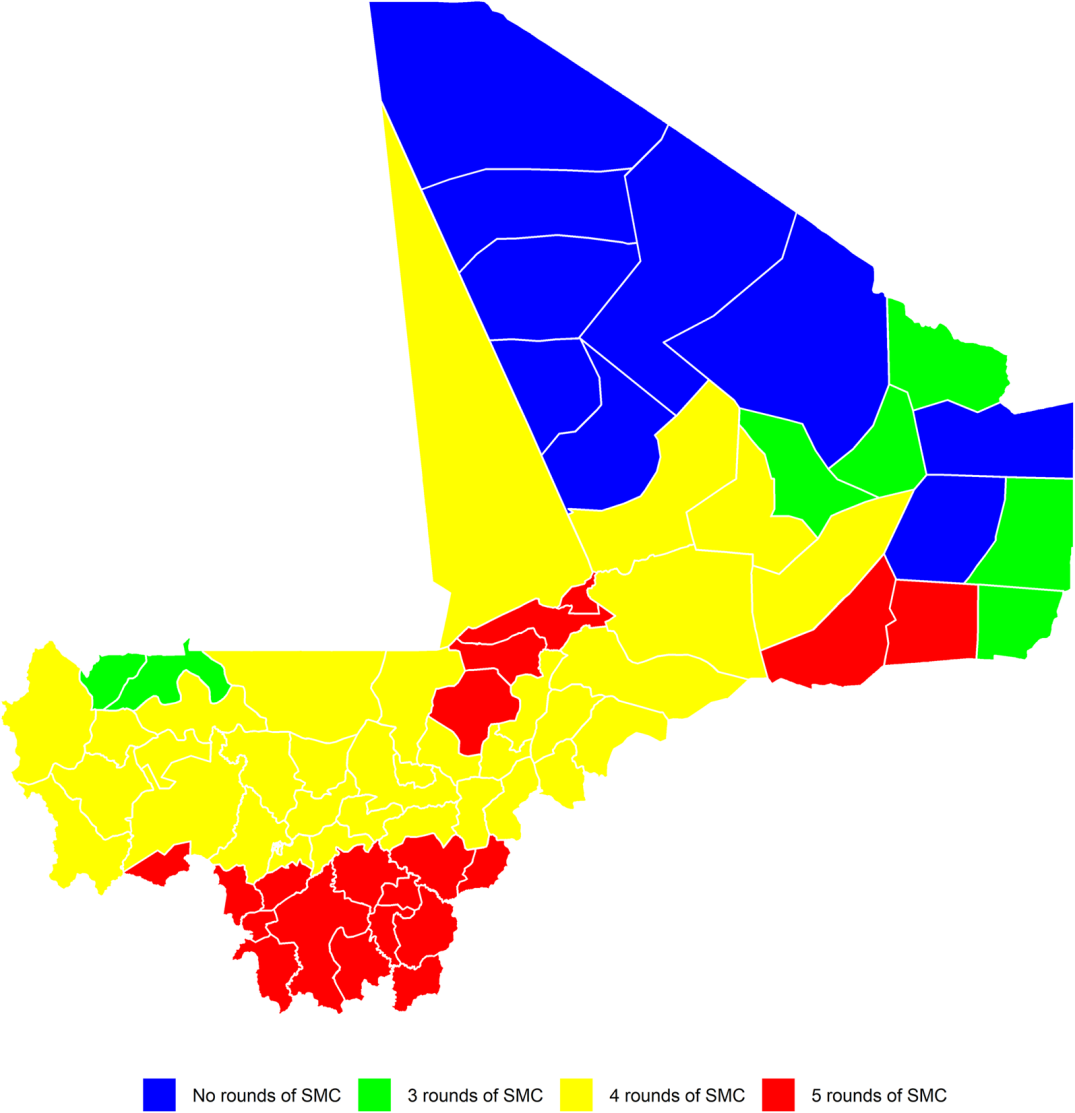
Number of cycles of seasonal chemoprevention needed in each health district based on the duration and seasonality of malaria transmission. Colour scale represents the required number of cycles. SMC, Seasonal malaria chemoprevention

Our analysis showed that the current strategy of conducting four cycles of SMC between July and October is appropriate in 39 of the 75 health districts. In 36 of these health districts, the number of cycles of SMC should be adapted to the duration and seasonality of malaria transmission. Specifically, 20 health districts should receive five cycles due to bimodal transmission and/or a long high transmission season, seven should receive three cycles due to a short high transmission season and nine should be excluded from SMC interventions due to irregular seasonality and/or low seasonal transmission (Fig. 4).

### Additional cost incurred and number of malaria cases, hospitalisations and deaths prevented annually using the best of the two predictive approaches

Based on an average unit cost of 0.7€, the overall cost of implementing SMC interventions in the 75 health districts using App-A would be 11,965,070€, compared to 11,404,705€ for the current approach. The additional cost incurred would be 560,365€, which represents less than 5% of the current cost.

**Fig. 5:**
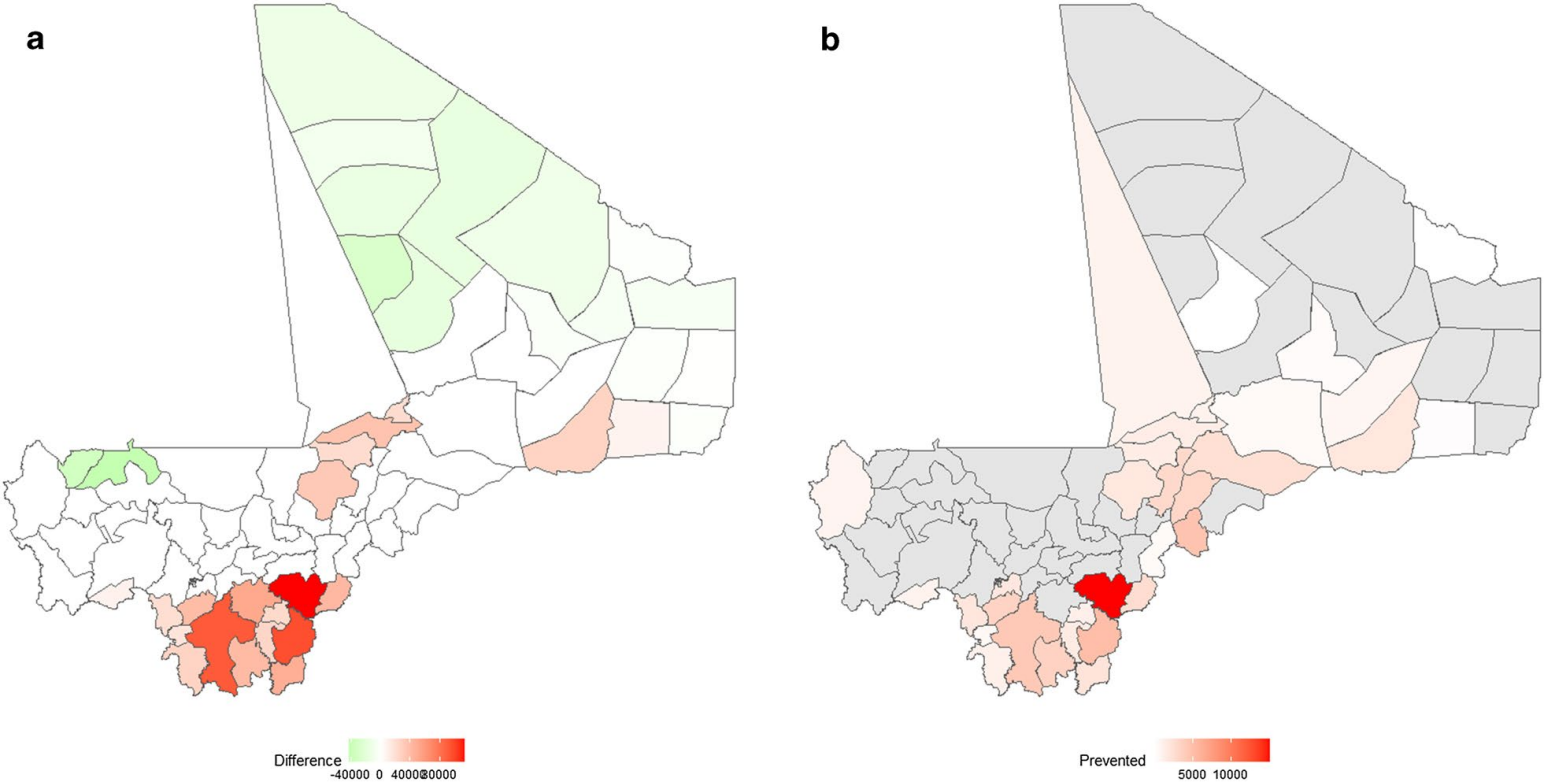
Maps showing the additional cost that would be incurred and the number of cases that would be prevented annually using App-A. **a** Additional cost incurred per health district, with the colour scale representing the difference between the cost of using the current approach and the cost of using App-A. **b** Number of malaria cases prevented per health district, with the colour scale representing the estimated number of cases that would be prevented using App-A

Using the best of the two approaches (App-A) would prevent 43,819 cases, 1943 hospitalisations and 70 deaths in children under 5 years (Fig. 5) annually.

## Discussion

Based on the results of this study, we propose a predictive approach for tailoring the timing and number of cycles of SMC in all health districts of Mali based on sub-national epidemiological surveillance and rainfall data. Our analysis shows that over the 2014–2019 study period, the onset of the rainy season varied between W17 (April) in the south and W34 (July) in the north along an environmental gradient. The onset of the high transmission season followed a similar pattern, with the high transmission season beginning in the Sahelian and Sudanese-Guinean zones one or two months earlier than in the Saharan zone. The median lag between the onset of the rainy season and the onset of the high transmission season ranged from 5 to 12 weeks, and the duration of the high transmission season varied between 3 and 6 months. Our findings suggest that the current malaria control strategy could be adapted in nearly half of Mali’s health districts in order to yield additional morbidity and mortality reductions. Using our proposed approach (App-A) would prevent 43,819 cases, 1943 hospitalisations and 70 deaths in children under 5 years of age (Fig. 5) annually for a minimal additional cost (560,365€).

The current malaria control strategy of the NMCP consists in delivering four cycles of SMC each year during the high transmission season across the 75 health districts of Mali. The timing and number of cycles of SMC were selected, respectively, based on the average onset of the high transmission season (from July to November) and the average duration of the high transmission season (4 months) [13]. Because this strategy does not account for the spatio-temporal heterogeneity of malaria transmission in Mali, some health districts receive SMC long before or long after the onset of the high transmission season, which likely compromises the global efficacy of SMC.

Our findings confirm that an average of four cycles of SMC is needed across the country. However, they also indicate that the effectiveness of SMC interventions could be improved by adapting the timing and number of cycles in 36 of the 75 health districts. Specifically, the number of cycles should be increased to five in health districts with bimodal transmission (i.e. in flooded areas or areas next to dams) and/or a long high transmission season; this finding is in line with results reported by Tagbor et al. which showed that the malaria burden could be reduced by 50% in a region of Ghana with a long high transmission season if ≥ 5 cycles of SMC were distributed annually [41]. In contrast, those health districts with irregular seasonality and/or low seasonal transmission (i.e. without cases for several weeks) appear to benefit little from SMC interventions. These health districts, all of them located in the arid north, could be excluded from the SMC program. Interestingly, Noor et al. [49] reached a similar conclusion in a study using seasonality, an age-structured population, urbanisation and malaria endemicity to identify health districts suitable for SMC targeting in Sahelian countries.

In 2019, the District of Bamako was excluded from the SMC program, as the 2018 DHS reported a low prevalence of malaria in children aged 6–59 months [2]. However, this survey was carried out during the SMC intervention and thus at a time when children were protected from malaria infection, suggesting that the prevalence of malaria was underestimated. Our study observed a high incidence of malaria in the general population of the District of Bamako, indicating that the city may need to be reintegrated in the SMC program. Indeed, studies have shown that the persistence of malaria in the general population can lead to epidemic resurgence, with severe cases in children [43, 44]. Moreover, the absence of premunition in the population of health districts with low seasonal transmission is known to increase the risk of epidemics [45]. These concerns are supported by a recent study showing that the incidence of malaria in children aged 6–59 months nearly doubled in the District of Bamako between 2018 and 2021 [46]. At the very least, the peri-urban area of Bamako should be reintegrated in the SMC program, as indicated by a meta-analysis of urban malaria in sub-Saharan Africa, which found the entomological inoculation rate to be sixfold higher in peri-urban areas (45.8) than in city centres (7.1) [47].

Sub-national tailoring of SMC interventions is now recommended by the WHO as part of the High Burden High Impact Initiative. In Senegal, SMC interventions have been tailored to the onset and duration of the rainy season since 2015. Interventions are initiated 1 month after the onset of the rainy season, with three cycles delivered in the regions of Kolda, Sédhiou and Tambacounda and four cycles delivered in the region of Kédougou [48]. Considering that the lag between the onset of the rainy season and that of the high transmission season is highly variable, our approach for predicting the onset of the high transmission season based on rainfall data allows for an even more refined tailoring of SMC interventions.

The additional cost of using our proposed approach was estimated by multiplying the average unit cost of SMC [14] by the number of target children in all health districts, a method used by Noor et al. [49] for the population-based estimation of malaria drug needs in sub-Saharan Africa in 2015–2020 [50]. The estimated additional cost is relatively low (560,365€) because the exclusion of health districts with irregular seasonality and/or low seasonal transmission would compensate for the increase in the number of cycles in health districts with bimodal transmission and/or a long high transmission season. In practice, this cost would likely be even lower because the health districts to be excluded are all located in the north of the country, where a very low population density leads to greater transport needs (including vehicle rental) and therefore to a higher unit cost of SMC. In this regard, the unit cost of SMC has also been shown to vary greatly in other Sub-Saharan countries. In Senegal, where the average unit cost (0.5€) is similar to that in Mali (0.7€) and covers roughly the same expenses, the unit cost in small health posts with few target children was found to be almost fivefold higher (2.5€) than the average unit cost [51]. In Ghana, the unit cost of SMC (per child receiving at least the first dose of four courses) has been shown to vary greatly depending on the mode of delivery (from 4.2€ for delivery by veterinary health workers to 4.5€ for delivery by consultation nurses and to 5.1€ for delivery by Expanded Programme on Immunisation nurses) [52]. To further reduce the cost of our proposed approach, the number of distribution days per cycle could be reduced from 5 to 4 days. As it turns out, some of Mali’s neighbouring countries (Senegal, Burkina Faso and Niger) have been distributing SMC over 3–4 days from the start [49].

In Mali, epidemiological surveillance data are mainly provided by the public and para-public sectors, as the rate of healthcare seeking in the private sector (including for specialised care) is very low at 3%. Indeed, private health care not developed to any great extent in the country, with 70% of private facilities located in the District of Bamako [53, 54]. Moreover, the vast majority of Malians prefer to seek care in the public and para-public sectors, where malaria testing and treatment for severe cases are free of charge. Currently, 26% of private healthcare facilities enter their malaria data directly in the DHIS2 sysem. Other private facilities send their data to community health centres and district hospitals, which in turn enter these data into the platform.

Monitoring activities in Mali have improved the quality of epidemiological surveillance data in recent years. In 2020, the data completeness rate was 98% (compared to 94.7% in 2016 and 76% in 2014) and the data promptness rate was 70.2% [33]. However, data quality has improved mainly in the south of the country. In the northern health districts, which suffer from political insecurity and low internet and telephone coverage, the completeness rate and the promptness rate were 60% and 42%, respectively, in 2020 [33]. These figures raise the possibility of underreporting in the Saharan zone. In the future, the quality of routine malaria data could be improved in this zone by adapting epidemiological surveillance to the local context. Should the low incidence of malaria transmission in the northern health districts be confirmed, malaria control could be strengthened by switching from weekly to daily epidemiological surveillance and by performing SMC interventions on a case-by-case basis. This approach has proven to be successful in the neighbouring Maghreb countries, which now have sporadic transmission and are considered to be malaria-free [55, 56].

The rainfall data used in our study were collected through remote sensing. Their quality could be compromised by poor satellite visibility, especially in the north where sand storms are common. However, several epidemiological studies have shown the relevance of using remote sensing rainfall data in the absence of observational data [57–59]. In particular, one study used remote sensing rainfall data to estimate malaria seasonality, populations at risk and areas to be targeted by SMC in Sahelian and sub-Sahelian countries [42]. In Mali, a recent study conducted in the northern health district of Diré found no difference between observational and remote sensing rainfall data [24], suggesting that the rainfall data used in our study are reliable. This reliability is supported by studies conducted in other malaria endemic countries, which reported lags between the onset of the rainfall season and the onset of the high transmission season that are consistent with our results [24, 60–62].

Given the variability of rainfall in the Sahelian zone [25, 63], some authors have suggested collecting rainfall data over several years for the planning of malaria control interventions in the area [64, 65]. This strategy has already shown its efficacy for agricultural production in the Gourma zone of Mali [66, 67]. It was also specifically recommended for the epidemiological surveillance of malaria in a study conducted in the Sahelian zone of Niger (Zinder) [68]. The predictive approach proposed in our study accounts for the high variability of rainfall over time and can therefore contribute to improving malaria epidemiological surveillance in Mali.

The main limitation of our study is that we used routine malaria data, which do not perfectly reflect the epidemiological situation on the ground. As mentioned earlier, however, the implementation of periodic reviews by the NMCP has considerably improved the quality of malaria data in recent years, especially in the southern health districts. Moreover, to correct for underreporting in the northern health districts, we collected malaria data from local reports when these were missing from the DHIS2. Another limitation of our study is that the number of preventable malaria cases, hospitalisations and deaths was calculated for children under 5 years of age instead of children aged 3–59 months, the latter being the age range selected for SMC interventions in Mali. Yet, it is unlikely that the efficacy of our approach was overestimated given that malaria is rare in children aged under 3 months. Lastly, entomological data were not included in our analyses because these are only available for a few sites in Mali. We compensated for the absence of entomological data by using rainfall data, which have been shown to correlate with malaria vector distribution and abundance [69]. This allowed us to propose a simple and effective approach for predicting the onset of the high transmission season in Mali.

Our proposed approach for tailoring the timing and number of cycles of SMC interventions at the operational scale could improve malaria control and reduce the malaria burden in Mali for a minimal additional cost. Future studies using high-quality routine data could further refine our approach by providing detailed estimations of additional costs in selected locations and/or by determining the effects of temperature, vegetation [70], vector abundance and insecticide resistance on the dynamics of malaria transmission [71, 72]. Our approach could thus be extended to other malaria endemic African countries with similar characteristics.

## Conclusion

The study proposes a simple and effective predictive approach for tailoring SMC interventions in Mali based on epidemiological surveillance and rainfall data. Future studies should be conducted to validate this approach with a view to extending it to other African countries with similar characteristics.

## Data Availability

The data and background maps are available on request.

## Abbreviations

ACT: Artemisinin-based combination therapy
CHIRPS: Climate Hazards Group InfraRed Precipitation with Station
DHS: Demographic and Health Survey
DHIS2: District Health Information Software version 2
DNP: National Population Directorate
GPHC: General Population and Housing Census
IRS: Indoor residual spraying
LLIN: Long-lasting insecticidal net
NMCP: National Malaria Control Program
RDT: Rapid diagnostic test
SMC: Seasonal malaria chemoprevention
